# Dissolution Behavior of M5 Cladding in Hydrofluoric–Nitric Mixed Acid

**DOI:** 10.3390/ma17235771

**Published:** 2024-11-25

**Authors:** Ying Chen, Yandong Sun, Yang Bai, Ziqian Zhao, Zheng Wei, Fang Liu, Zhongwei Yuan, Taihong Yan, Weifang Zheng

**Affiliations:** China Institute of Atomic Energy, Beijing 102413, China; 18801064330@163.com (Y.C.); sunyandong7@163.com (Y.S.); youngby@126.com (Y.B.); 15811373835@163.com (Z.Z.); wzheng401@163.com (Z.W.); liufang40131@163.com (F.L.); yuanzw99@163.com (Z.Y.); yanthcn@163.com (T.Y.)

**Keywords:** M5 cladding, HF-HNO_3_ mixed acid, dissolution kinetics, passivation layer

## Abstract

M5 cladding has emerged as a prominent fuel cladding material due to its excellent corrosion resistance. The dissolution behavior of M5 cladding is critical in both the initial cleaning stage and the reprocessing of spent fuel cladding. This study investigated the dissolution of M5 cladding in hydrofluoric–nitric (HF-HNO_3_) mixed acid at varying concentrations. When the HF concentration exceeds 0.5 mol/L, the addition of strong oxidizing HNO_3_ significantly reduces the dissolution rate. Moreover, HNO_3_ effectively inhibits the HF-induced corrosion pitting, lowering surface roughness to 0.812 μm at a 1:5 ratio of HF:HNO_3_. In addition, a surface structural analysis reveals the dissolution mechanism of M5 cladding. The β-Nb precipitated in the mixed acid was oxidized to stable Nb_2_O_5_ by HNO_3_ while the M5 matrix surface was continuously oxidized to ZrO_2_. This passivation layer inhibits further dissolution, slowing the process and enhancing the uniformity of M5 cladding.

## 1. Introduction

Zirconium (Zr) alloys are used as the fuel cladding material in most water reactor-type nuclear reactors because of their small neutron absorption cross-section and favorable corrosion resistance [1,2,3,4]. In recent years, niobium (Nb)-modified zirconium alloys have been developed to enhance their mechanical properties [5,6]. More importantly, the formation of a stable passivation layer under oxidizing conditions has been shown to improve long-term corrosion resistance [7,8,9,10,11]. Given its superior performance in demanding conditions like high burnup rates, elevated temperatures, and low pH environments, M5 (Zr-Nb alloy) has gradually surpassed traditional Zircaloy-4 (Zr-Sn alloy) as the mainstream choice for pressurized water reactors in nuclear power plants [12,13,14,15,16].

Impurities tend to be adhered to the surface of newly fabricated zirconium cladding, so it needs to be cleaned before use [17,18,19]. In addition, the surface dissolution of spent fuel cladding is employed to reduce α waste (radioactive waste containing alpha-emitting radionuclides, such as uranium and plutonium), providing an efficient decontamination method [20,21]. The uniform gradient dissolution of spent fuel cladding can obtain the radial distribution of radionuclides, supplying data support for the recovery of zirconium cladding [22,23,24]. Therefore, both the cleaning of zirconium cladding and the reprocessing of spent fuel cladding rely on dissolution technology.

Numerous studies have revealed the dissolution behavior of zirconium alloys in acidic solutions via dissolution kinetics [25,26]. Vander Wall proposed that the dissolution rate of zirconium is solely dependent on the concentration of unionized hydrofluoric acid, with the diffusion of hydrofluoric acid molecules across the boundary layer serving as the rate-determining step [27]. Smith observed that the initial dissolution rate of zirconium exhibits a first-order dependence on the HF concentration when HF ranges from 10^−3^ to 10^−1^ mol/L [28]. James reported deviations from this linear rate law when the F^−^ concentration surpasses 0.5 mol/L [29]. Gu found that with an increasing HNO_3_ concentration, the reaction products of a zirconium alloy in mixed acids gradually transition into an amorphous state [30]. Research by R. Klein further indicates that at HF concentrations above 0.5 mol/L, the dissolution rate of zirconium becomes markedly sensitive to the HNO_3_ concentration, and when the HNO_3_ concentration exceeds 0.4 mol/L, the dissolution rate decreases as the HNO_3_ concentration increases [31].

Although the dissolution behavior of Zircaloy-4 in a mixed acid of hydrofluoric and nitric (HF-HNO_3_) has been well studied [30,31,32,33,34,35], relatively few investigations have focused on the dissolution behavior of Nb-rich M5 cladding in mixed acid. The reduced dissolution rate of Zircaloy-4 in such mixed acid is typically attributed to the formation of oxide and fluoride passivation layers, which effectively inhibit the dissolution reaction [36,37,38,39]. However, for M5 cladding, the specific chemical composition of the passivation layer, its formation mechanism, and the detailed processes by which it slows down dissolution remain unclear. Additionally, the presence of Nb in M5 cladding and its influence on the dissolution behavior need to be further investigated.

Here, the aim of this work is to investigate the dissolution behavior of M5 cladding in HF-HNO_3_ mixed acid with different ratios of hydrofluoric to nitric acid. The dissolution kinetics of M5 cladding was established by continuous weight loss measurements. It was shown that the HNO_3_ fraction inhibited the dissolution rate at HF concentrations above 0.5 mol/L. Specifically, the M5 cladding was uniformly dissolved in the optimized ratio (1:5) of mixed acid, and the surface roughness was reduced to 0.812 μm. By analyzing the changes in the surface micromorphology and chemical components during the dissolution process, we elucidated the process of the formation of the passivation layer and proposed a decelerating mechanism for the dissolution of the M5 cladding in the mixed acid. This study provides data and theoretical support for the decontamination process of Nb-containing cladding in pickling and post-treatment.

## 2. Materials and Methods

### 2.1. Materials

The zirconium alloy used here was M5 cladding supplied by the industry China Nuclear North Fuel Components Co. Ltd. (Baotou, China) (inner diameter: 8.4 mm, outer diameter: 9.54 mm), possessing a composition of Nb 1, Fe 0.03, O 0.14 wt%, and Zr. The samples are cladding tube segments, cut to 10 mm length by machining. For surface morphology observation, the segments were cut and flattened to produce sheets and then obtained samples were wet ground through P100, P400, and P1200 silicon carbide abrasive papers. All samples were cleaned in acetone, then rinsed in ethanol and dried before use. Their initial mass was measured with a 0.1 mg accuracy. The acids used here include HNO_3_ (65 wt%) and HF (40 wt%). An acid mixture of HNO_3_ and HF in a specific ratio was prepared. Due to the corrosive nature of HF, all experimental manipulations were carried out in polytetrafluoroethylene containers.

### 2.2. Dissolution Testing

The experiments were carried out on M5 cladding samples at a constant temperature bath (25 °C) with stirring at 500 r/min. Care was taken to ensure that the stirrer rotor did not contact or knock the samples, as this could alter the reaction surface area. Acid solutions of varying concentrations were prepared, with HNO_3_ ranging from 0 to 5 mol/L and HF from 0.25 to 2.5 mol/L. The amount of dissolved M5 cladding per gram was well below the saturation threshold, and the dissolution of contained trace elements such as Nb and Fe did not affect the mass. At specific intervals (e.g., 50 s, 300 s, 600 s, 900 s, and 1800 s), samples were carefully removed, thoroughly rinsed with distilled water to eliminate any residual acid to prevent further surface reactions, and then gently dried with absorbent paper followed by naturally drying. Each sample was immediately weighed, and the recorded weights across varying acid concentrations and time intervals were subsequently used to calculate dissolution rates. For the next time point, a completely fresh M5 cladding sample was used, which was then immersed in the new acid solution for the next specific duration. This process was repeated for each subsequent data point, ensuring that each sample underwent only one immersion and treatment. The dissolution rates were calculated based on the weight differences across various acid concentrations and time intervals, allowing for analysis of the effects of acid concentrations on the dissolution kinetics of M5 cladding. The detailed experimental procedures and a schematic illustrating the process are provided in the Supplementary Materials Figure S1.

The mass loss rate was calculated using Equation (1):

(1) $$mass\;loss\;\left(\%\right)=\frac{\Delta m}{m_{0}}\times 100\%$$

where Δ*m* represents the difference obtained by subtracting the instantaneous mass from the initial mass and *m*_0_ represents the initial mass of the sample (g).

The dissolution rate was calculated using Equation (2) [24]:

(2) $$v\;(g{cm}^{-2}\;s^{-1})=\frac{\Delta m}{At}$$

where *v* represents the dissolution rate (an average rate over each interval), *A* represents the sum of the inner and outer surface areas of the sample (cm^2^), and *t* represents the dissolution time (s).

### 2.3. Characterization

The microstructure of the M5 sample after HF and mixed acid dissolution was characterized and measured using an optical microscope (Leica DM2700 M, Wetzlar, Germany). The 3D surface topographies of the M5 samples treated with HF and mixed acid were analyzed with the white light interferometer (Zygo, Newview 7100, Middlefield, CT, USA). The properties and chemical structures of the black powder and black powder based on M5 (BP/M5) were determined by an X-ray diffractometer (Bruker D8 Advance, Billerica, MA, USA). Microstructural analyses of the original M5, HF-treated M5, and mixed-acid-treated M5 samples were performed using a field-emission scanning electron microscope (Hitachi SU8020, Tokyo, Japan) with an acceleration voltage of 5.0 kV. The BP/M5 sample underwent both SEM and compositional analysis using the attached energy-dispersive X-ray spectroscopy (EDS) accessory. The Raman spectrum of the BP/M5 sample was measured with a Raman spectrometer (Renishaw inVia Raman microscope, 532 nm laser, power 1 mW, Wotton-under-Edge, UK). The composition of the elements and their chemical states were investigated using X-ray photoelectron spectroscopy (Thermo Scientific ESCALAB 250Xi, Waltham, MA, USA) and an Al Kα source.

## 3. Results

### 3.1. Dissolution Rate

**Figure 1:**
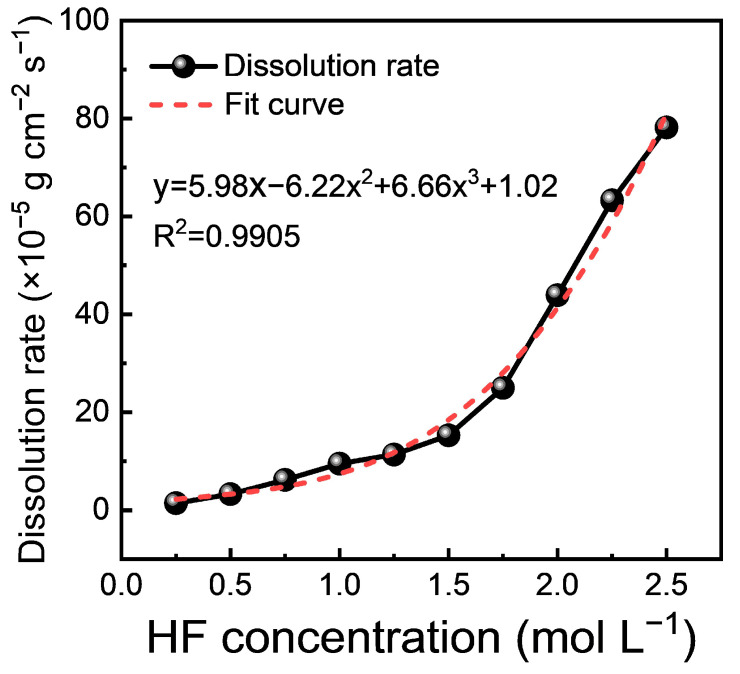
Dissolution rate of M5 cladding in HF solution at concentrations of 0.25–2.5 mol/L.

As shown in Figure 1, the dissolution rate of the M5 cladding is extremely sensitive to the HF concentration. Under pure HF conditions, the reaction rate accelerates significantly as the HF concentration increases. The dissolution rate as a function of HF concentration is described by the following polynomial Equation (3):

y = 5.98x − 6.22x^2^ + 6.66x^3^ + 1.02 (3)

where *x* represents the HF concentration and *y* represents the dissolution rate. The high *R*^2^ value of 0.9905 indicates that this polynomial equation effectively captures the trend in the experimental data, indicating a strong correlation between acid concentration and dissolution rate.

In the initial stage of dissolution, from approximately 0.25 to 1.0 mol/L, the dissolution rate increases almost linearly with the concentration, reflecting a direct correlation between the acid concentration and the reactivity of the M5 cladding surface. As the concentration continues to increase, the dissolution rate shifts to a nonlinear regime, with a steep rise observed from approximately 1.0 to 2.5 mol/L. At higher concentrations, the slope becomes even steeper, indicating an accelerated dissolution process likely due to increased HF ionization and enhanced surface reactivity at higher concentrations. Furthermore, the presence of a cubic term (6.66x^3^) in the equation indicates that the relationship between the dissolution rate and acid concentration is not simply linear or quadratic. This suggests that the dissolution behavior is complex and may involve multi-step reactions or surface chemistry changes, highlighting the importance of further investigating structural and compositional changes during dissolution to understand the underlying mechanisms.

**Figure 2:**
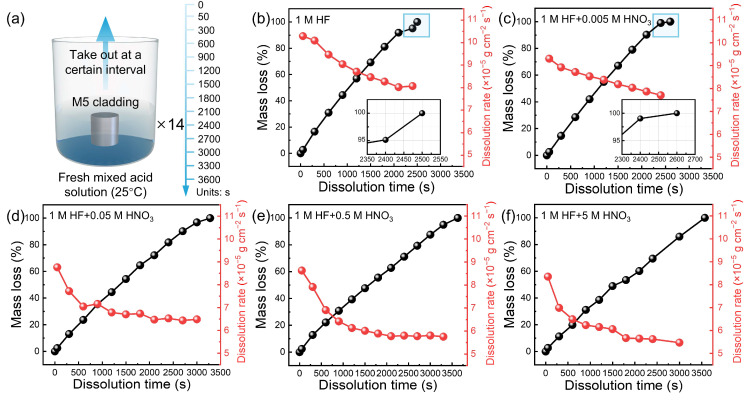
(**a**) Dissolution of M5 cladding in HF-HNO_3_ mixed acid solutions at 25 °C. Mass loss and dissolution rate of M5 cladding in HF-HNO_3_ mixed acid (**b**) 1:0, (**c**) 1:0.005, (**d**) 1:0.05, (**e**) 1:0.5, and (**f**) 1:5 (HF:HNO_3_ ratio).

The dissolution of M5 claddings was carried out in HF-HNO_3_ mixed acid at 25 °C in a constant temperature bath. To ensure consistency, 14 identical solutions from the same batch were used, with M5 cladding removed at specific intervals for data collection (Figure 2a). The kinetic curves show the dissolution of M5 cladding in mixed acid with HF-HNO_3_ ratios of 1:0, 1:0.005, 1:0.05, 1:0.5, and 1:5 (Figure 2b–f). The dissolution rate is relatively fast in 1 mol/L HF solution and 1:0.005 mixed acid, with the rate remaining above 8 × 10^−5^ g cm^−2^ s^−1^ throughout the reaction (Figure 2b,c). However, as the concentration of HNO_3_ increases, the dissolution rate in mixed acid (1:0.05, 1:0.5, and 1:5) gradually slows down (Figure 2d–f). The average reaction rate decreased from 8.9 × 10^−5^ g cm^−2^ s^−1^ in the pure HF solution to 5.2×10^−5^ g cm^−2^ s^−1^ in the 1:5 mixed acid, representing a 41.57% reduction in rate. 

To facilitate a direct comparison of the dissolution rates across the five groups, we compared the dissolution rate at 50 s, 900 s, and 1800 s. As shown in Table 1, the dissolution rate gradually decreased with the addition of HNO_3_ over the same time intervals. In the pure HF solution, the dissolution rate at 50 s was 10.29 × 10^−5^ g cm^−2^ s^−1^, while in the 1:5 mixed acid, it decreased to 8.35 × 10^−5^ g cm^−2^ s^−1^. Although the dissolution rate decreases significantly at 50 s, the inhibitory effect at this early stage is notably weaker than that observed at later times, such as 900 s and 1800 s.

In the comparison between the 1:0 and 1:5 mixed acid at 900 s, the dissolution rate in the pure HF solution was 9.04 × 10^−5^ g cm^−2^ s^−1^, whereas in the 1:5 mixed acid, it decreased to 6.23 × 10^−5^ g cm^−2^ s^−1^, a reduction of 2.81 × 10^−5^ g cm^−2^ s^−1^. By 1800 s, the dissolution rate of the 1:5 mixed acid further declined to 5.67 × 10^−5^ g cm^−2^ s^−1^, showing an additional decrease of 0.56 × 10^−5^ g cm^−2^ s^−1^ compared to 900 s. This trend indicates that the dissolution rate decreases over time, with the highest rate observed in the pure HF solution and the lowest in the 1:5 mixed acid. Furthermore, the data highlight that the presence of HNO_3_ in a 1 mol/L HF solution inhibits the dissolution rate.

The reaction of Zr with different ratios of HF-HNO_3_ was analyzed based on Supplementary Materials. The addition of HNO_3_ to the acid mixture changes the reaction products and increases the activation energy of the reaction, ultimately rendering HNO_3_ relatively inert to Zr [30,37], and it is conducive to the formation of a protective oxide film on the surface of the alloy to inhibit corrosion [6]. Therefore, it is essential to study the kinetics and compositional changes in M5 cladding during the mixed acid dissolution process, which would provide a more comprehensive explanation of the dissolution behavior of M5 cladding.

**Figure 3:**
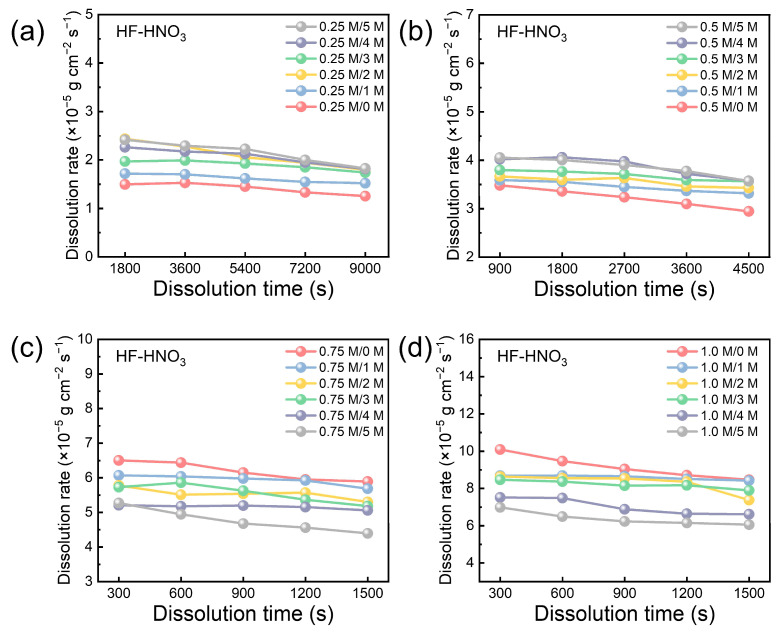
Dissolution rate of M5 cladding in HF-HNO_3_ acid mixtures solution with varying HNO_3_ concentrations (0–5 mol/L) and HF concentrations of (**a**) 0.25, (**b**) 0.5, (**c**) 0.75, and (**d**) 1 mol/L.

To establish a suitable range of the gradient dissolution rate, the dissolution of the M5 cladding was conducted until the unruptured cladding structure was used as the reaction endpoint. Figure 3 shows the dissolution rate trend of the M5 cladding across various HF (0.25–1 mol/L) versus HNO_3_ (0–5 mol/L) ratios. At a constant HNO_3_ concentration, the 1.0 mol/L HF solution exhibited the highest initial dissolution rate, while the 0.25 mol/L HF solution showed the lowest. The red line in Figure 3 highlights the effect of HF when considered as a single variable, demonstrating that the dissolution rate of M5 cladding generally decreases over time. Extrapolated HF consumption, based on the amount of Zr loss, suggests that the theoretical reaction rate at the end of the reaction should be approximately 0.5–1 × 10^−5^ g cm^−2^ s^−1^ lower than the initial rate, consistent with the observed decrease of about 0.5 × 10^−5^ g cm^−2^ s^−1^.

The effect of HNO_3_ concentration on the dissolution rate was significantly different, varying with the mixed acid systems containing HF concentration ranges. In mixed acid with 0.25–0.5 mol/L HF, the dissolution rate was lowest in pure HF solutions, and the addition of HNO_3_ slightly increased the dissolution rate (Figure 3a,b). Specifically, the average dissolution rate increased by 0.74 × 10^−5^ g cm^−2^ s^−1^ in the 0.25:5 mixed acid compared to 0.25:0 and by 0.64 × 10^−5^ g cm^−2^ s^−1^ in the 0.5:5 mixed acid compared to the 0.5:0. Conversely, in mixed acid with 0.6–1 mol/L HF, the dissolution rate was highest in pure HF solutions, while the addition of HNO_3_ significantly decreased the dissolution rate (Figure 3a,b and Figure S2). The average dissolution rate decreased by 1.42 × 10^−5^ g cm^−2^ s^−1^ in the 0.75:5 mixed acid compared to the 0.75:0 and by 2.77 × 10^−5^ g cm^−2^ s^−1^ in the 1:5 mixed acid compared to the 1:0.

**Figure 4:**
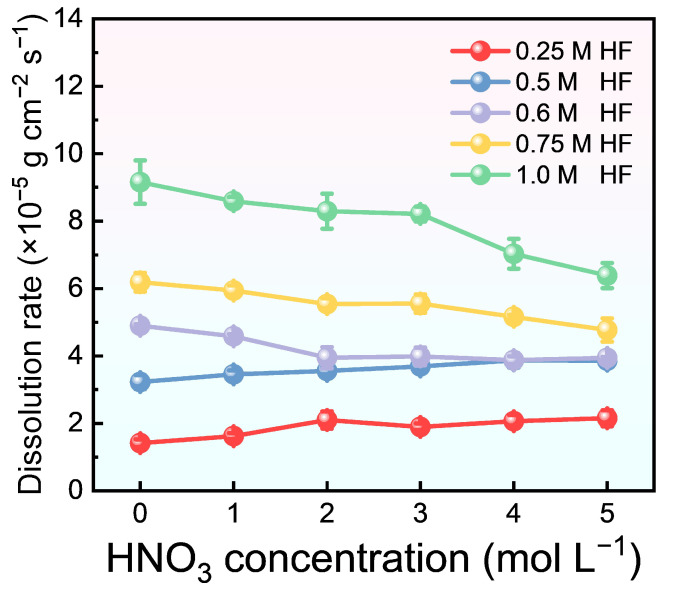
Summary of dissolution rate for M5 cladding in HF-HNO_3_ mixed acid across various ratios.

To provide a more intuitive comparison of the dissolution rate trends, Figure 4 summarizes the relationship between the dissolution rate and varying concentrations of HF and HNO_3_. The error bars in the figure represent the standard deviation of the measured dissolution rate at different time points. Analysis of the effects of HNO_3_ on dissolution kinetics reveals that its addition to 0.25 mol/L and 0.5 mol/L HF solutions enhances the dissolution rate to some extent. However, at an HF concentration exceeding 0.5 mol/L, the addition of HNO_3_ significantly inhibits the dissolution rate. The rate-controlling step is the diffusion of HF molecules through the zirconium boundary layer [31,39]. In mixed acid systems with HF concentrations below 0.5 mol/L, HNO_3_ can inhibit HF ionization, thereby promoting the dissolution. Conversely, at HF concentrations above 0.5 mol/L, the literature suggests that HNO_3_ alters the dissolution reaction products [30], which in turn restricts HF diffusion through the zirconium boundary layer, resulting in a reduced reaction rate.

### 3.2. Surface Morphology and Uniformity

**Figure 5:**
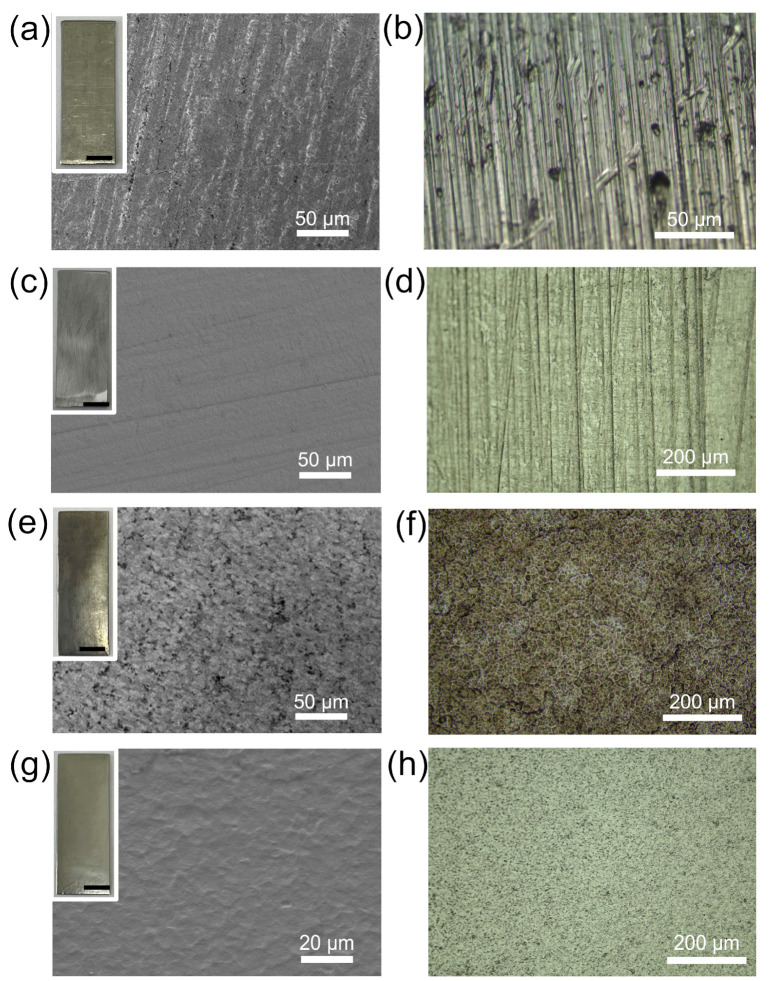
(**a**) SEM and (**b**) OM images of the raw M5; (**c**) SEM and (**d**) OM images of the polished M5; (**e**) SEM and (**f**) OM images of the M5 after treatment with HF (1 mol/L) solution; (**g**) SEM and (**h**) OM images of the M5 after treatment with HF-HNO_3_ (1:5) mixed acid. Insets: photographs of corresponding samples. All scale bars: 5 mm.

To compare the surface morphology of M5 under different dissolution conditions, rinsed M5 samples were analyzed for their microscopic features. Figure 5a presents a scanning electron microscope (SEM) image of the raw M5, with the inset revealing a dull and lusterless surface. This appearance can be attributed to the natural oxidation of metallic zirconium in air, leading to the formation of an oxide layer approximately 2–5 μm thick [40]. Additionally, rolling marks from the manufacturing process are visible on the surface of the raw M5. Figure 5b presents an optical microscope (OM) image of the raw M5, showing minor dents likely introduced during processing or transportation, but the surface structure remains intact. The images of the polished M5 show that, despite the polishing treatment, subtle polishing marks are still visible on the surface (Figure 5c,d). Similar to the raw M5, the polished M5 retains an intact crystal structure. Therefore, in subsequent acid treatments, the raw M5 was utilized directly after a cleaning step for dissolution.

Figure 5e illustrates a SEM image after treatment with the 1 mol/L pure HF solution, highlighting the undulations and peaks characteristic of the surface at the microscopic level. The inset reveals the formation of a black, porous hydride film on the surface. Figure 5f presents an OM image showing fine grains and granular precipitation phases, indicative of more severe corrosion of M5 by fluoride ions.

In the HF-HNO_3_ mixed acid, a layer of dense black powder formed on the surface of the M5 cladding, which could be easily rinsed off to reveal the underlying metal matrix (Figure S3). Figure 5g shows a SEM image of the M5 sample treated with the 1:5 mixed acid, with the inset revealing a much smoother surface with a mirror-like luster. As shown in Figure 5h, the mixed acid treatment gives the M5 surface a more uniform and finer texture. This treatment appears to reduce differential corrosion and give a more consistent structural profile. This appearance is attributed to the protective black powder layer formed during the dissolution process, which effectively shields the zirconium matrix from direct corrosion by mixed acid. Even without being pre-treated by polishing, the microscopic morphology of the substrate appears relatively flat, maintaining an intact and unbroken surface structure.

**Figure 6:**
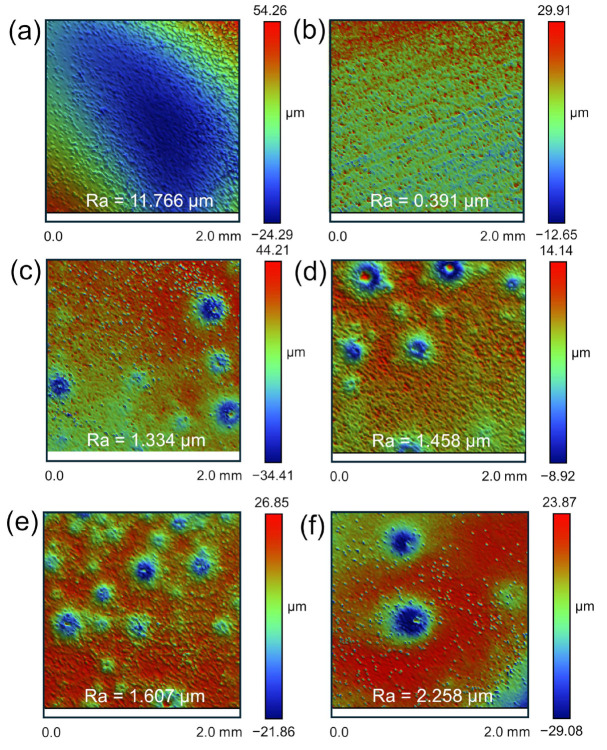
Three-dimensional morphology and surface roughness of (**a**) raw M5 sample and (**b**) M5 sample pre-treated by polishing and grinding. Three-dimensional morphology and surface roughness of M5 samples dissolved in HF solution (**c**) 0.25, (**d**) 0.5, (**e**) 0.75, and (**f**) 1 mol/L.

We analyzed the effect of the HF concentration on the surface roughness of the M5 samples. The initial surface roughness of the untreated samples was found to exceed 10 μm, displaying significant unevenness (Figure 6a). Therefore, to ensure consistency in the sample matrix before testing, the M5 samples were pre-treated through polishing and sanding, reducing the surface roughness to below 0.4 μm (Figure 6b).

The M5 samples were exposed to the HF solution at concentrations of 0.25, 0.5, 0.75, and 1 mol/L until their mass was reduced by half, ensuring the consistent dissolution of the zirconium matrix across all samples. After treatment with the 0.25 mol/L HF solution, the surface roughness increased from 0.319 μm to 1.334 μm (Figure 6b,c). Based on the height and surface topography observed in the interferometric images, the treated samples display prominent etch pits. This is primarily attributed to the formation and escape of hydrogen bubbles during the reaction. The escape of these bubbles causes the exfoliation of the products in the weakly adhered areas, promoting the diffusion of hydrofluoric molecules along the zirconium boundary layer and resulting in localized corrosion and a subsequent increase in surface roughness [41,42]. In addition, the formation of these pits can be linked to several factors reported in the literature, including the action of highly corrosive acid media, the precipitation of minor inclusions such as ZrF_4_ and ZrO_2_, and the localized rupture of the zirconia passivation film [7,43].

As the HF concentration increased, the surface roughness rose from 1.334 μm to 2.258 μm, with the diameter of the etch pits also enlarging correspondingly (Figure 6c–f). This is attributed to the rapid formation of larger bubbles on the sample surface at higher HF concentrations. The escape of these bubbles exacerbates pit formation, and the continuous dissolution process leads to the gradual merging of pits, eventually forming larger and deeper pits, which significantly increases the surface roughness. This phenomenon indicates that the high HF concentration has a pronounced erosive effect on the zirconium surface, making it prone to localized pitting and thereby affecting the overall corrosion uniformity of the samples.

**Figure 7:**
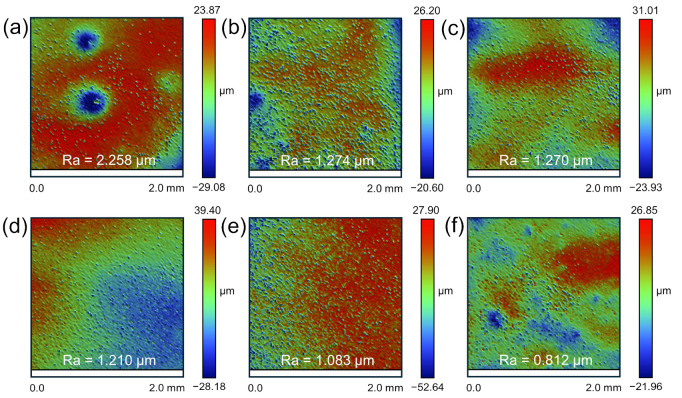
Three-dimensional morphology and roughness of M5 samples dissolved in HF-HNO_3_ mixed acid solutions: (**a**) 1:0, (**b**) 1:1, (**c**) 1:2, (**d**) 1:3, (**e**) 1:4, and (**f**) 1:5.

Figure 7a–f show the surfaces of the M5 samples after treatment with mixed acid at different ratios: 1:0, 1:1, 1:2, 1:3, 1:4, and 1:5. Compared to samples treated with a pure HF solution (Figure 7a), the mixed acid treatments significantly improved the surface roughness, reducing it by more than 1 μm, with no etch pits observed (Figure 7b–f). The presence of HNO_3_ mitigated the aggressive corrosion by HF, reduced bubble formation, and prevented the formation of etch pits caused by the localized spalling of reaction products. Specifically, in mixed acid with an HF concentration of 1 mol/L, as the HNO_3_ concentration increased, the surface became progressively smoother, with roughness decreasing to 1.274, 1.27, 1.21, 1.08, and 0.81 μm. Thus, higher concentrations of HNO_3_ in the mixed acid not only slow the surface corrosion but also prevent pitting, thereby reducing surface roughness.

### 3.3. Analysis of the Passivation Layer

**Figure 8:**
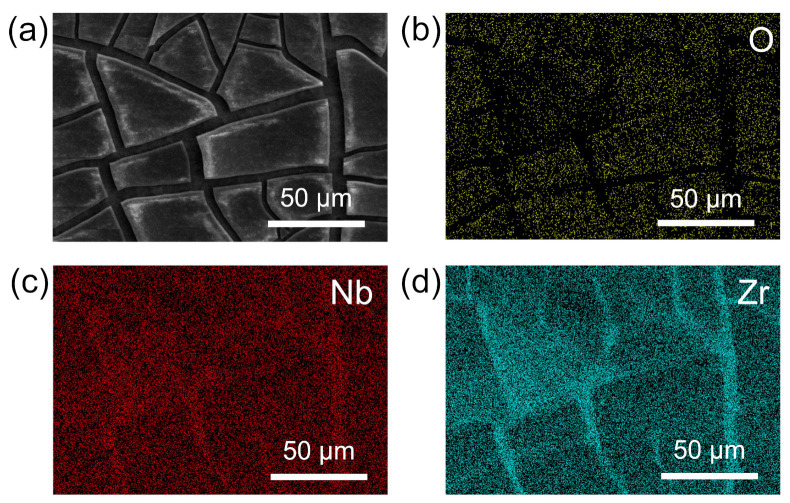
(**a**) SEM image of BP/M5 surface. Elemental mappings of (**b**) O, (**c**) Nb, and (**d**) Zr.

To provide a baseline for comparison, the surface of the polished M5 sample was analyzed in Figure S4, showing the SEM image (Figure S4a) and the elemental distribution mapping of Zr, Nb, and O (Figure S4b–d). The results indicate that in the raw M5, the Zr and Nb elements are evenly distributed within the M5 matrix. To investigate the effect of black powder formation on the dissolution process, the microstructure and elemental distribution of the M5 sample covered with black powder (BP/M5) were analyzed by SEM. As shown in Figure 8a, the black powder cracks in the natural environment and covers the M5 matrix surface in a flake-like manner. The EDS mappings of the O, Nb, and Zr elements are displayed in Figure 8b–d. The yellow dots indicate the distribution of O, showing a clear concentration in the blocky regions of the black powder, with only a minimal presence at the cracks (Figure 7b). The red dots show the location of Nb, which is relatively uniformly distributed without obvious aggregation (Figure 8c). The blue dots depict the distribution of Zr with a high density in the cracks (Figure 8d). The micromorphology of the surface after the black powder is rinsed off from the metal matrix is shown in Figure S5a, with the EDS mapping of O, Nb, and Zr (Figure S5b–d). Beneath the black powder, the cleaned surface primarily consists of metallic Zr and Nb in the M5 matrix. These analytical results suggest that the black powder gradually accumulates on the zirconium matrix surface during the dissolution process. This black powder contains not only β-Nb but also metal oxides, which collectively contribute to slowing down the dissolution process.

Furthermore, the localized over-distribution of Zr outside the crack was further analyzed. Figure S6 shows a photograph of BP/M5, with some white powder visible on the right side. Figure S7a displays the SEM image of the white powder after the acid dissolution solution dried. The elemental distribution maps in Figure S7b–d illustrate the distribution of Zr, F, and N in the white powder residue, which helps explain the observed localized Zr concentration. This high zirconium content is likely due to residual acid remaining on the surface during the preparation of BP/M5, which formed the white powder residue upon drying. Based on the reaction equations in Supplementary Materials, ZrF_4_(H_2_O) and NH_4_ZrF_5_ tend to accumulate in specific areas, resulting in the observed zirconium enrichment.

It is widely accepted that the slower dissolution rate of zirconium alloys in HF-HNO_3_ acid mixtures is due to the formation of protective oxide and fluoride films on the surface. For the special M5, Nb tends to segregate into very small particles within the alloy, known as second-phase particles (SPPs) [6,44,45]. During the dissolution of Zr-Nb alloys in mixed acid, the zirconium matrix dissolves more rapidly than the SPPs. This difference in dissolution rate leads to the release of numerous fine black particles, which accumulate and adhere to the surface of the zirconium matrix, forming a dense black powder layer.

**Figure 9:**
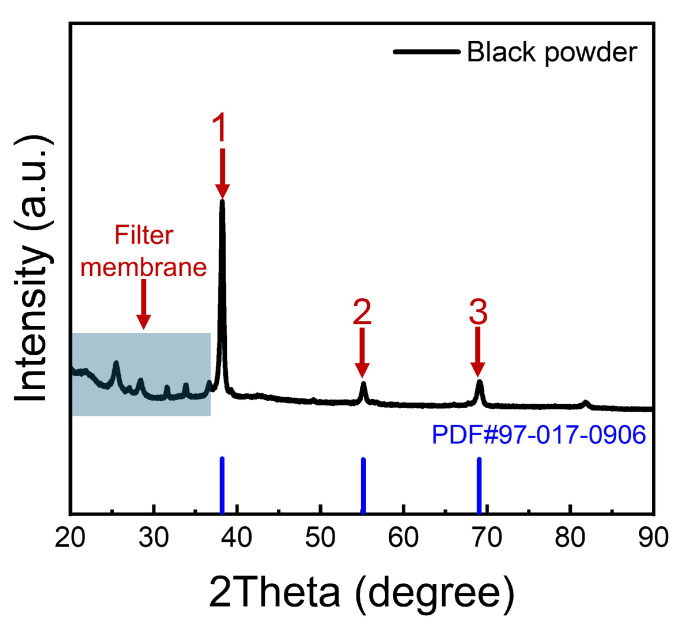
XRD pattern of filtered black powder on the filter membrane.

The X-ray diffraction (XRD) pattern of the filtered black powder is shown in Figure 9. After subtracting the background peaks from the filter paper, three prominent diffraction peaks were observed at 38.215°, 55.153°, and 69.079°, which correspond to the characteristic peak positions of Nb with a body-centered cubic structure. Specifically, these peaks are associated with different crystal planes of Nb: 2θ = 38.215° corresponds to the (110) plane, 2θ = 55.153° to the (200) plane, and 2θ = 69.079° to the (211) plane. These peaks confirm the presence of the β-Nb phase with a body-centered cubic crystal structure (PDF#97-017-0906), which is consistent with the characteristics of the β-Nb phase.

**Figure 10:**
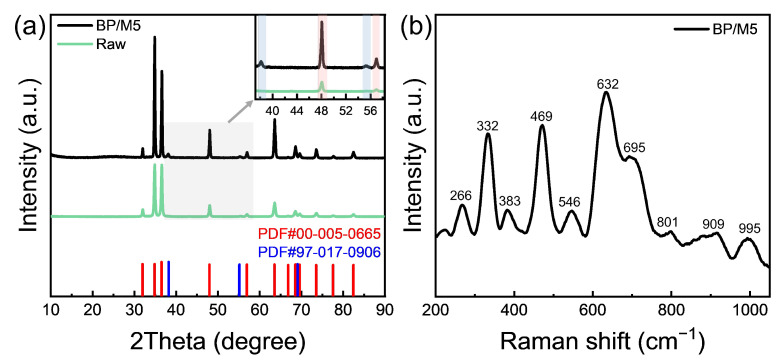
(**a**) XRD pattern of BP/M5, the magnified section highlights a slight peak of Nb (×1.5). (**b**) Raman spectra of BP/M5.

The surface chemical composition changes during the mixed acid treatment of M5 were further investigated by phase analysis. The XRD patterns of both the raw M5 sample and the BP/M5 sample were compared with standard reference diffractograms. Due to the detection limitations of XRD, the oxide layer was too thin to be detected. The XRD pattern of the raw M5 sample matches perfectly with the reference pattern for pure zirconium (PDF#00-005-0665) (Figure 10a, green line). For the BP/M5 sample, the matrix mainly exhibits zirconium (PDF#00-005-0665). The inset, highlighted in blue, reveals the presence of a small amount of Nb (PDF#97-017-0906), primarily originating from SPPs (β-Nb) (Figure 10a, black line) [46].

Raman spectra are sensitive to the structure and chemical bonding of metal oxides, particularly in the metal–oxygen stretching mode region. Figure 10b illustrates distinct Raman peaks at 266, 332, 383, 469, 546, 632, 695, 801, 909, and 995 cm^−1^ for the BP/M5 sample, with characteristic peaks corresponding to the oxides of Zr and Nb based on their respective wave number ranges. The peaks can be attributed to specific vibrational modes: the 200–400 cm^−1^ region corresponds to the bending mode of Nb–O–Nb while the 400–800 cm^−1^ bands are associated with the symmetric and vibrational modes of Nb–O–Nb bonds, and the high-frequency Raman bands in the 900–1200 cm^−1^ range are primarily due to the terminal Nb=O stretching mode [47,48]. Additionally, Zr–O bond bending vibrations appear in the range of 300 cm^−1^ to 500 cm^−1^ while stretching vibrations are concentrated in the 500–800 cm^−1^ range. Since the vibrational modes of Zr–O bonds in ZrO_2_ are prominent within the 300–800 cm^−1^ range, higher intensity peaks are observed in this region [44,49].

Based on the above analysis, the metal oxides consist of Nb_2_O_5_ and ZrO_2_. During the dissolution process of M5 in the HF-HNO_3_ mixed acid, β-Nb is continuously precipitated from the surface, while both β-Nb and Zr in the M5 matrix are oxidized by the high concentration of HNO_3_ to form stable oxides. In view of the inhibition of the dissolution process in mixed acid, we refer to the combination of precipitated SPPs (β-Nb) along with the oxides of Zr and Nb as a passivation layer.

**Figure 11:**
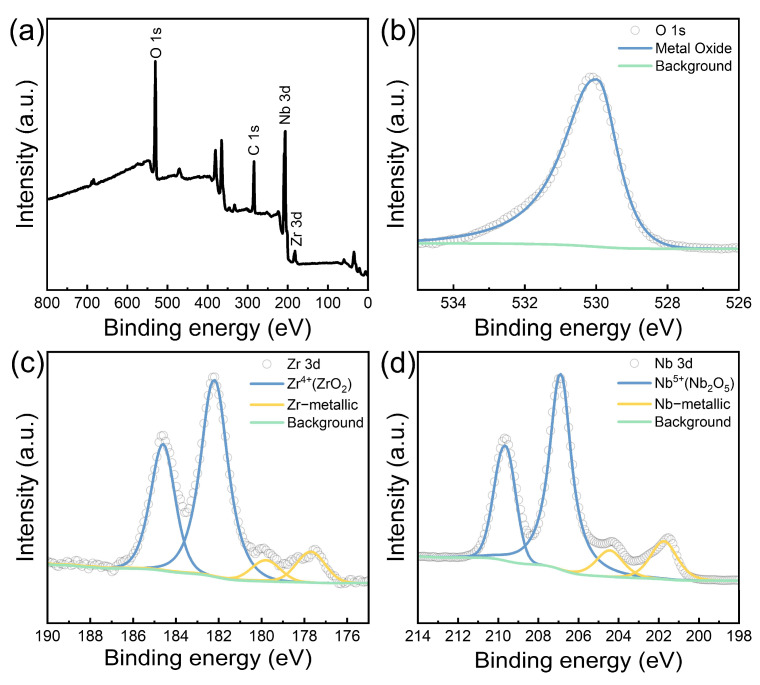
(**a**) XPS survey spectrum of BP/M5. High-resolution XPS scan spectra over (**b**) O 1s, (**c**) Zr 3d, and (**d**) Nb 3d peaks of BP/M5.

To further investigate the compositional structure of the passivation layer, the BP/M5 sample was analyzed using X-ray photoelectron spectroscopy (XPS). The XPS survey spectrum reveals characteristic peaks corresponding to C 1s, O 1s, Nb 3d, and Zr 3d (Figure 11a). The binding energy values for each element in its respective chemical state (oxide or metal) are listed in Table 2. The C 1s peak corresponded to the unavoidable contamination of carbon formed during sample handling, with the peak at 284.6 eV used as an internal reference for calibrating absolute binding energy. The O 1s peak at 529.9 eV originated from oxygen bonded to metals (Figure 11b). The deconvoluted Zr 3d spectrum revealed two sets of doublet peaks at the respective binding energy values (Table 2), corresponding to Zr existing in both the +4 oxidation state (ZrO_2_) as well as in metallic form (Figure 11c). Similarly, the deconvoluted high-resolution spectrum in the Nb 3d region (Figure 11d) shows peaks at binding energies corresponding to the metallic state (201.7 eV and 204.4 eV) and the +5 oxidation state (206.8 eV and 209.6 eV) [8,50]. After adjusting for the respective sensitivity factors, the intensity of the Nb 3d peak is noticeably higher than that of the Zr 3d peak, suggesting that Nb in the passivation layer is primarily located on the outer surface of the Zr.

The ratio of Zr and Nb oxides to metals is calculated from the area under the corresponding deconvolution peaks. (Table 2). The results indicate that 79.6% of Nb is present in the oxide state while 83.6% of Zr is in the oxide state, with the remainder in the metallic state. Based on the compositional distribution within the passivation layer during the dissolution process, it can be inferred that β-Nb particles were continuously precipitated on the M5 matrix during the continuous dissolution in mixed acid. The accumulated β-Nb outer layer was rapidly oxidized to Nb_2_O_5_ while the exposed fresh M5 matrix was concurrently oxidized to ZrO_2_ by the high concentration of HNO_3_. Hence, these findings clearly demonstrate that the passivation layer structure includes Nb_2_O_5_, β-Nb, and ZrO_2_.

## 4. Discussion

**Figure 12:**
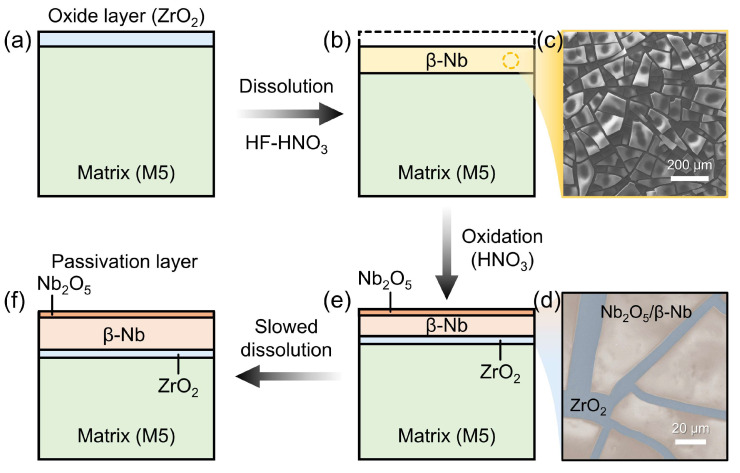
Schematic representation of dissolution mechanism of M5 cladding in HF-HNO_3_ mixed acid. (**a**) Raw M5 cladding. (**b**) M5 cladding treated with the mixed acid solution. (**c**) SEM image of the β-Nb on the surface of the M5 cladding. (**d**) SEM image of the oxide products on the surface of the M5 cladding. (**e**) Oxidation of the surface precipitate of the M5 cladding. (**f**) Formation of a passivation layer that slows the dissolution rate.

During the dissolution of M5 cladding in HF-HNO_3_ mixed acid, the HF concentration directly determines the dissolution rate, while the presence of HNO_3_ significantly affects the dissolution rate and the uniformity surface [31]. To deeply understand the mechanism with the associated changes in surface composition and morphology of the M5 sample, a schematic diagram depicting the dissolution mechanism is shown in Figure 12.

The M5 cladding readily forms a thin oxidized layer when exposed to air, which is quickly dissolved in the initial stage of dissolution, leaving subsequent analyses unaffected (Figure 12a). As dissolution progresses, β-Nb second-phase particles in the M5 matrix begin to precipitate [46], forming a layer of black powder covering the surface of the matrix (Figure 12b). Microstructural observations of the BP/M5 layer reveal that the β-Nb layer serves as an effective barrier, limiting the inward diffusion of the solution medium (Figure 12c).

As the proportion of HNO_3_ in the mixed acid increases, the β-Nb precipitated layer comes into direct contact with the strong oxidizing acid, leading to the oxidation of β-Nb to stable Nb_2_O_5_ (Figure 12e) [8]. The oxidation process of β-Nb causes the accumulation of localized stress in the oxidized film of zirconium alloys, which facilitates the generation of microcracks (Figure 12d). The microcracks create pathways for the mixed acid to diffuse, thus continuing the dissolution process. However, the exposed M5 matrix at the cracks is also subjected to oxidation by HNO_3_, leading to the continuous formation of a ZrO_2_ layer on the surface of the matrix.

In the later stages of the dissolution process, the accumulation of precipitated β-Nb and metal oxides continues as dissolution and oxidation progress, leading to a gradual thickening of the passivation layer (Figure 12f). The outer oxide layer of the passivation layer primarily results from the oxidation of precipitated β-Nb, while the inner oxide layer is mainly formed by the oxidation of the M5 matrix. The limited diffusion rate of the acid through the passivation layer results in a reduced concentration of HF near the substrate interface. Additionally, the strong oxidizing effect of HNO_3_ in the mixed acid prevents the formation of pitting. This not only slows down the dissolution but also makes the dissolution process more uniform, thereby enhancing the overall consistency and controllability of the dissolution.

The results of this study further corroborate the formation of a protective passivation layer on the surface of zirconium alloys in an oxidizing acid environment. Jayaraj demonstrated that high-entropy alloys containing Zr formed a protective passivation layer in HNO_3_ and NaF solutions, finding that the addition of Nb and Ta significantly enhanced the corrosion resistance of the alloy [8,51]. Similarly, Yao investigated the formation of Nb_2_O_5_ oxide films in Zr-Nb alloys, emphasizing their critical role in improving the stability of alloys [46]. These findings align with our observations during the dissolution process. However, in contrast to previous studies, our research primarily focuses on the mechanisms underlying the observed slowdown in the dissolution rate of the M5 cladding during mixed acid dissolution. During the dissolution and oxidation processes, β-Nb, Nb_2_O_5_, and ZrO_2_ gradually accumulate, forming a protective passivation layer. This passivation layer effectively restricts acid diffusion within the layer, ultimately slowing the dissolution rate of the M5 cladding. Our study provides further insight into the factors contributing to the reduced dissolution rate of M5 cladding in mixed acid, offering a novel perspective for a more comprehensive understanding of the dissolution process.

## 5. Conclusions

In summary, the dissolution behavior of M5 cladding in various HF-HNO_3_ mixed acid concentrations was investigated. This study examined the impact of mixed acid on the microscopic morphology and surface roughness of M5 cladding. Additionally, the mechanism of dissolution was analyzed based on changes in surface microstructure and the composition of the passivation layer formed. These findings provide theoretical insights for the cleaning of M5 cladding and for achieving the uniform dissolution of spent fuel cladding. The key findings include the following:

(1) The dissolution kinetics of M5 cladding indicate that the dissolution rate is positively correlated with the HF concentration in HF-HNO_3_ mixed acid. However, when the HF concentration exceeded 0.5 mol/L, the dissolution rate was significantly reduced due to the strong oxidizing effect of HNO_3_.
(2) Increasing the HNO_3_ concentration in the mixed acid mitigates the aggressive erosive action of HF, reduces pitting corrosion, and enhances dissolution uniformity. Under optimized acid conditions (1:5), the M5 cladding achieves uniform dissolution, with surface roughness reduced to 0.812 μm.
(3) During dissolution, SPPs (β-Nb) precipitate as a black powder. The outer layer of the precipitate is partially oxidized to stable Nb_2_O_5_ by a high concentration of HNO_3_, while the underlying M5 matrix continues to oxidation to ZrO_2_. As a result, the surface of the M5 cladding forms a passivation layer consisting of SPPs (β-Nb), Nb_2_O_5_, and ZrO_2_.
(4) The dissolution behavior of M5 cladding in mixed acid is primarily influenced by the formation of the passivation layer. As dissolution and oxidation proceed, Nb oxide and β-Nb continuously accumulate in the outer region of the layer while Zr oxide forms on the surface of the M5 matrix. Meanwhile, the passivation layer restricts acid diffusion to the M5 matrix, thereby slowing down the dissolution of the M5 cladding and improving the overall uniformity of the dissolution process.

In summary, future research should further investigate the effects of varying temperature conditions on dissolution rates and passivation layer formation. Conducting dissolution experiments under different temperature conditions will help reflect the impact of thermal effects on dissolution behavior in practical applications. Additionally, the applicability of the findings should be validated using spent fuel cladding samples to assess their practical effectiveness.

## Figures and Tables

**Table 1:** Dissolution rates of M5 cladding in mixed acid at 50 s, 900 s, and 1800 s time intervals.

Dissolution Rate (HF:HNO_3_ Ratio)	50 s (×10^−5^ g cm^−2^ s^−1^)	900 s (×10^−5^ g cm^−2^ s^−1^)	1800 s (×10^−5^ g cm^−2^ s^−1^)	Reduction Between 900 s and 1800 s (×10^−5^ g cm^−2^ s^−1^)
1:0	10.29	9.04	8.27	0.77
1:0.005	9.30	8.53	8.03	0.50
1:0.05	8.76	7.16	6.73	0.43
1:0.5	8.63	6.42	5.89	0.53
1:5	8.35	6.23	5.67	0.56

**Table 2:** Binding energies and concentrations of oxide and metallic states for elements in BP/M5.

Element	Photo Electron lines	Oxidation/Chemical State	Binding Energy (eV)	Atomic Concentration (%)
O	1s	O^2−^ (metal oxide)	529.9	100
Nb	3d5/2; 3d3/2	Nb^5+^ (Nb_2_O_5_)	206.8; 209.6	79.6
		Nb	201.7; 204.4	20.4
Zr	3d5/2; 3d3/2	Zr^4+^ (ZrO_2_)	182.1; 184.5	83.6
		Zr	177.6; 179.7	16.1

## Data Availability

The data presented in this study are available upon request from the corresponding author.

## Supplementary Materials

The following supporting information can be downloaded at https://www.mdpi.com/article/10.3390/ma17235771/s1: Note 1: Equations of the reaction [30]; Figure S1: Schematic of the dissolution process and sample treatment; Figure S2: Dissolution rate of M5 cladding in HF-HNO_3_ acid mixtures solution; Figure S3: Original image of BP/M5 cladding and BP/M5 cladding after rinsing [8]; Figure S4: SEM image and elemental mappings of the polished M5 surface; Figure S5: SEM image and elemental mappings of the surface after rinsing off the black powder from BP/M5; Figure S6: Photograph of M5 after treatment with mixed acid; Figure S7: SEM image and elemental mappings of white powder residue.
