# Supplementary material for: Dissolution Behavior of M5 Cladding in Hydrofluoric–Nitric Mixed Acid
Source: Materials (Basel). 2024 Nov 25;17(23):5771. doi: 10.3390/ma17235771 (PMC11642294; doi:10.3390/ma17235771)
Supplement: Supplementary file 1 [file materials-17-05771-s001.zip › materials-3293824-supplementary.pdf]

Supplementary material for

Dissolution Behavior of M5 Cladding in Hydrofluoric–Nitric Mixed Acid

Ying Chen, Yandong Sun, Yang Bai, Ziqian Zhao, Zheng Wei, Fang Liu, Zhongwei Yuan, Taihong Yan and Weifang Zheng *

China Institute of Atomic Energy, Beijing 102413, China; 18801064330@163.com (Y.C.); sunyandong7@163.com (Y.S.); youngby@126.com (Y.B.); 15811373835@163.com (Z.Z.); wzheng401@163.com (Z.W.); liufang40131@163.com (F.L.); yuanzw99@163.com (Z.Y.); yanthcn@163.com (T.Y.)

* Correspondence: zhengwfcn@163.com

Table of Contents

1. Supplementary Method 3

2. Supplementary Note 4

3. Supplementary Figures 5

4. Supplementary References 11

1. Supplementary Method

Dissolution and treatment process

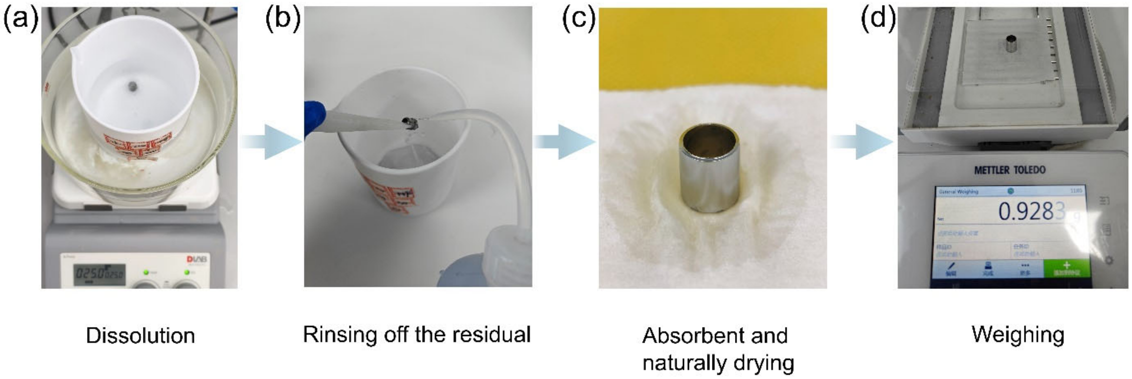

Figure S1. Schematic of the dissolution process and sample treatment. (**a**) Dissolution: M5 cladding samples are immersed in mixed acid solutions constant temperature bath (25°C), with stirring at 500 r/min. (**b**) Rinsing off the residual: After dissolution, the samples are thoroughly rinsed with distilled water to remove residual acid. (**c**) Absorbent and naturally drying: The samples are blotted with absorbent paper and then naturally dried. (**d**) Weighing: The dried samples are weighed to determine the mass loss, allowing the calculation of the dissolution rate under different conditions.

2.Supplementary Note

Supplementary Note 1. Equations of the reaction.

Equations (a–c) illustrate the reaction process for different proportions of hydrofluoric-nitric (HF-HNO_3) and the resulting formation of various zirconium compounds. As the proportion of HNO_3 increases, the solid reaction products of the zirconium alloy and mixed acid transition from $ZrF_4(H_2O)$ and $NH_4ZrF_5(H_2O)$ to $Zr(OH)_2(NO_3)_2(H_2O)$. Concurrently, the weight percentage of amorphous substances increases, while the weight percentage of crystalline products decreases [1].

$$Zr + 4HF + H_2O \rightarrow ZrF_4(H_2O) + 2H_2\uparrow \quad (a)$$

$$4Zr + 5HNO_3 + 17HF \rightarrow 3ZrF_4(H_2O) + NH_4ZrF_5 + 2N_2O_3\uparrow + 6H_2O \quad (b)$$

$$Zr + 4HNO_3 + (x-1)H_2O \rightarrow Zr(NO_3)_2(OH)_2x(H_2O) + 2N_2O_3\uparrow \quad (c)$$

2. Supplementary Figures

In light of the varying effects observed upon the addition of HNO_3 to 0.5 mol/L and 0.75 mol/L HF solutions, we further investigated the dissolution behavior in a 0.6 mol/L HF solution. As shown in Figure S2, the dissolution rate at varying HNO_3 concentrations is highest in the absence of HNO_3 and decreases as the HNO_3 increases. However, when the HNO_3 concentration exceeds 2 mol/L, there is no further significant decrease in the dissolution rate. Despite this plateau, HNO_3 continues to exert an overall inhibitory effect. Therefore, the addition of HNO_3 to HF solution with concentration above 0.5 mol/L results in sustained inhibition of the dissolution rate.

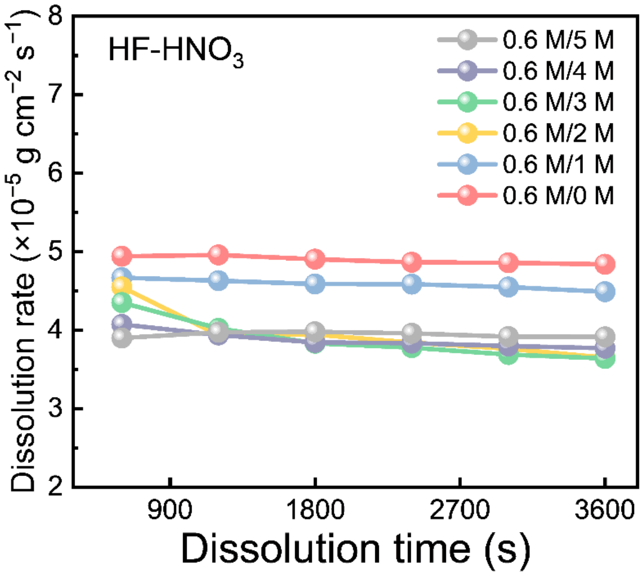

Figure S2. Dissolution rate of M5 cladding in HF-HNO_3 acid mixtures solution with varying HNO_3 concentrations (0−5 mol/L) and HF concentrations of 0.6 mol/L.

As shown in Figure S3, the left side depicts M5 cladding covered with black powder (BP/M5 cladding) after dissolution in HF-HNO_3 mixed acid [2]. This black powder forms a uniform and complete protective layer over M5 matrix. In contrast, the right side shows BP/M5 cladding after rinsing. where the black powder has been removed. The exposed metal matrix is smooth flat, free of pits, and exhibits a distinct luster.

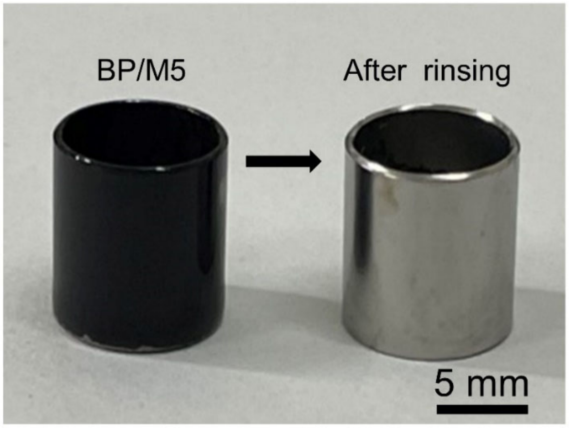

Figure S3. Original image of BP/M5 cladding and BP/M5 cladding after rinsing.

The surface micro-morphology of the polished M5 is shown in Figure S4a, clearly revealing the M5 matrix structure. Figures S4b−d present the elemental distribution maps of O, Nb, and Zr on the polished M5 surface, showing a uniform and dense distribution of these elements. The consistent dispersion of Nb and Zr indicates a stable composition across the polished M5 surface, reflecting the even integration of these metallic elements within the matrix.

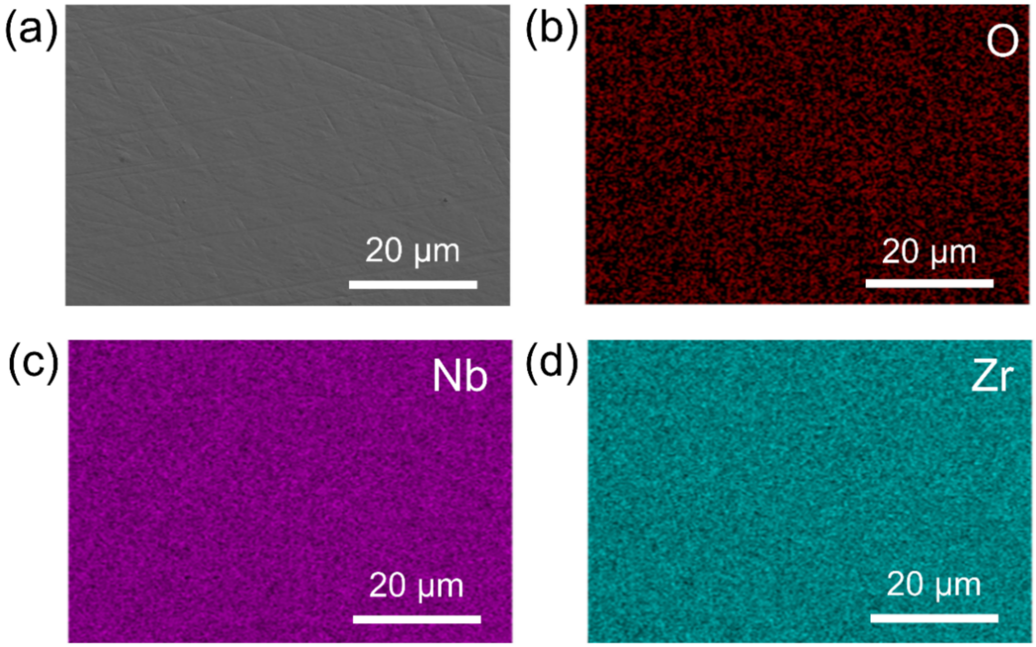

Figure S4. (**a**) SEM image of the polished M5 surface. Elemental mappings of (**b**) O, (**c**) Nb, and (**d**) Zr on M5 surface.

The micro-morphology of the surface after rinsing off the black powder from BP/M5 is shown in Figure S5a, revealing the underlying M5 matrix. The EDS mappings of O, Nb, and Zr elements are displayed in Figures S5b−d. The yellow dots indicate the distribution of O, which is relatively low in concentration. The red points show the location of Nb, exhibiting a uniform and dense distribution, similar to the Zr element indicated by the blue dots in Figure S5d. Overall, the elements are uniformly distributed on the surface, suggesting that the rinsed surface is primarily composed of Zr and Nb in their metallic states within the M5 matrix.

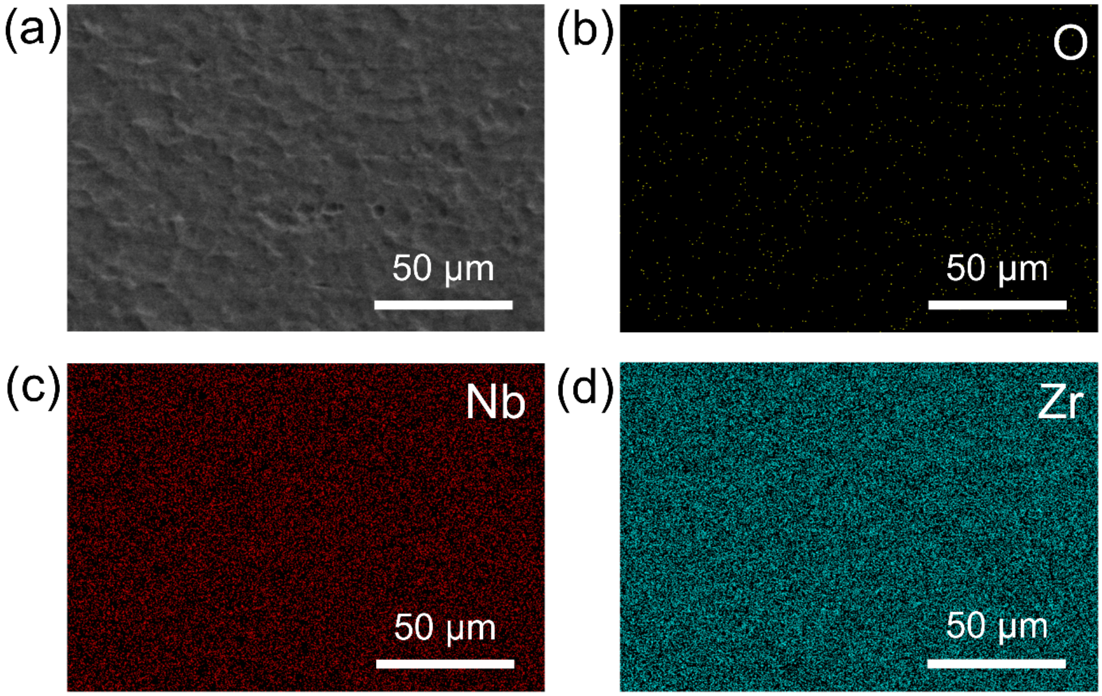

Figure S5. (**a**) SEM image of the surface after rinsing off the black powder from BP/M5. Elemental mappings of (**b**) O, (**c**) Nb, and (**d**) Zr on the rinsed BP/M5 surface.

In the preparation of BP/M5 samples, a challenge arises due to the presence of β-Nb attached to the M5 surface. During the process to remove residual acid, β-Nb can easily be washed away. To reduce the impact of acid on the black powder layer, we gently shook off residual acid from the surface. However, small amounts of acid remained, which dried to form a white powder residue. Figure S6 shows the M5 surface, with the left image displaying the ideal black powder, while the right image highlights the prominent white powder residue.

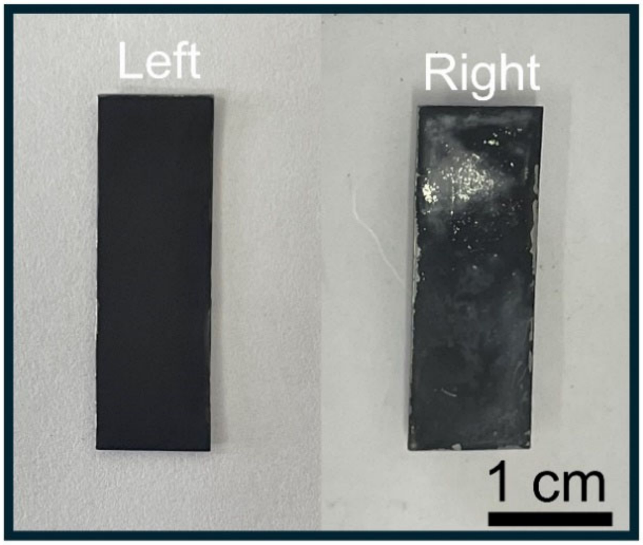

Figure S6. Photograph of M5 after treatment with mixed acid: the left side shows the BP/M5, and the right side shows the BP/M5 with white powder residue.

Figure S7a presents an SEM image of the white powder collected after dissolving the M5 sample and drying the acid dissolution solution. Figures S7b–d display the elemental distribution maps of Zr, F, and N in the white powder, showing a uniform distribution of these elements. Based on the elemental distribution of Zr in the EDS analysis and the reaction equations in Note 1, it can be inferred that the BP/M5 sample contains components such as $ZrF_4(H_2O)$ and NH_4ZrF_5.

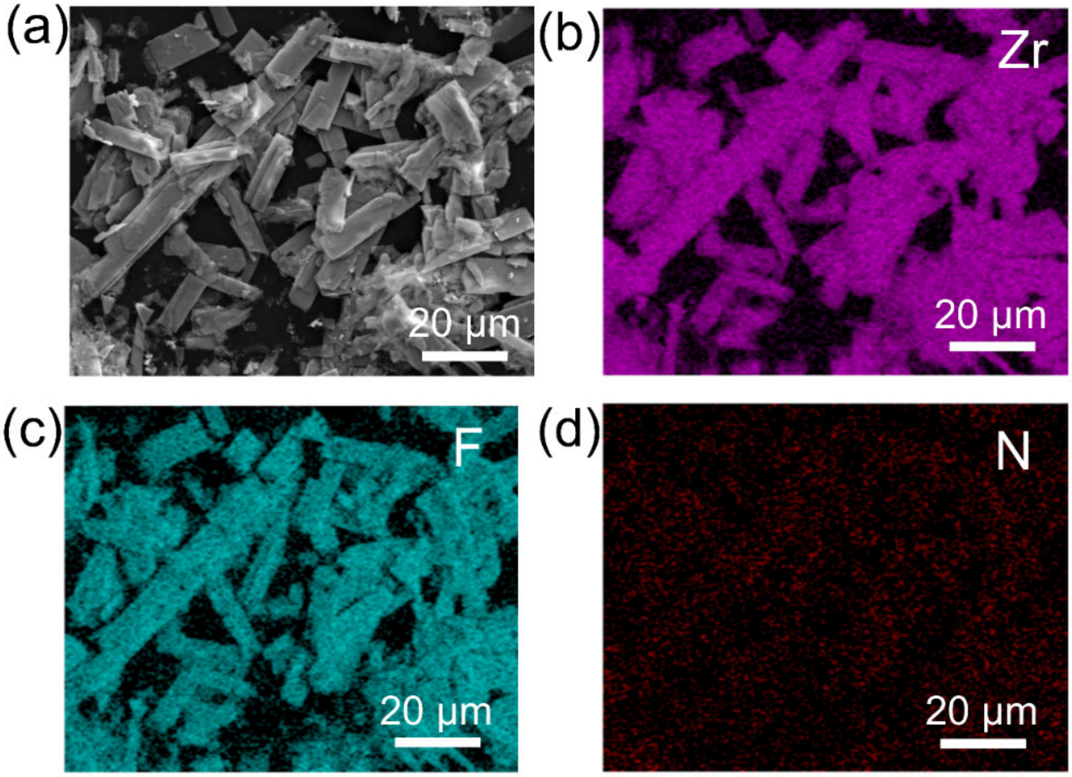

Figure S7. (**a**) SEM image of white powder residue. Elemental mappings of (**b**) Zr, (**c**) F, and (**d**) N on the white powder residue.

4. Supplementary References

[1] H. Gu, G. Li, C. Liu, F. Yuan, L. Zhang, L. Wang, X. Li, S. Wu, S. Peng, B. Gao, Characterization of the products obtained from the reactions of Zircaloy-4 with an acid mixture of concentrated HNO_3 and dilute HF with the aim of understanding pickle salts of zirconium alloys, RSC Advances, 6 (2016) 109815-109825.

[2] J. Jayaraj, C. Thinaharan, S. Ningshen, C. Mallika, U. Kamachi Mudali, Corrosion behavior and surface film characterization of TaNbHfZrTi high entropy alloy in aggressive nitric acid medium, Intermetallics, 89 (2017) 123-132.
